# Improving the health of African Americans in the USA: an overdue opportunity for social justice

**DOI:** 10.1186/s40985-016-0025-4

**Published:** 2016-10-03

**Authors:** Allan S. Noonan, Hector Eduardo Velasco-Mondragon, Fernando A. Wagner

**Affiliations:** 1 United States Public Health Services, Hunt Valley, Maryland, 21030 USA; 2 Touro University California College of Osteopathic Medicine, 1310 Johnson Lane, Vallejo California, 94592 USA; 3 Morgan State University School of Community Health and Policy, 4530 Portage Avenue Campus, 1700 E. Cold Spring Lane, Baltimore, MD 21251 USA

**Keywords:** Ethnicity and health, African Americans, Health disparities, Social justice, Social determinants of health

## Abstract

Using a modified social ecological model, we conducted a review of the literature and nationwide statistics on African American health. We discuss the main social determinants of health and main health disparities, risk factors, the leading causes of morbidity and mortality, and access to health services for blacks in the USA. The mechanisms through which social determinants, including racism, exert their deleterious effects on black health are discussed at the macro and individual levels. Incarceration and mental health care issues are highlighted as priorities to be addressed. African Americans remain the least healthy ethnic group in the USA, a somber legacy of years of racial and social injustice and a formidable challenge to equitable health care for all. Systemic causes of suboptimal black health require equally systemic solutions; positive trends in black health indicators seem to be driven by social development programs, economic investment in education, participation of African Americans in policy, and decision-making and expansion of access to health care.

## Background

In 1928, Louis Israel Dublin wrote “An improvement in Negro health, to the point where it would compare favorably with that of the white race, would at one stroke wipe out many disabilities from which the race suffers, improve its economic status and stimulate its native abilities as would no other single improvement. These are the social implications of the facts of Negro Health” [1]. This compelling assertion remains valid to date. The fact that the African American population is the least healthy ethnic group in the USA is not due to chance. The first African Americans were brought to the USA in chains as slaves. The transport itself from Africa to the New World remains one of the best examples of the ability of one sector of humanity to destroy the health of another. Estimates of the death rate of slaves during the infamous “middle passage” are wide ranging, from approximately 9 to 35 %. Slavery associated deaths were likely much higher [2, 3]

Once enslaved in what is now the USA, African Americans were forced to live in physical and social conditions in which their health had very little value. For more than 250 years, enslaved African Americans suffered physical, social, and mental brutalization. The end of slavery did not mean that African Americans could suddenly lead healthful lives. To the contrary, they have been subjected to systematic discrimination and oppression for the 150 years since slavery was abolished, and it continues nowadays. Healthwise, this history may be viewed as resulting in two outcomes. With so much suffering and early death, those who survived this subjection may be the strongest and most resilient members of this group. However, the history of slavery and the current racial discrimination this group continues to suffer clearly underlie the inexcusably poor health status of African Americans as a whole.

In 1984, Margaret Heckler, then Secretary of Health and Human Services (HHS), dissatisfied with the way health disparities were being reported to Congress, provided the first comprehensive review of health disparities endured by black and minority groups, compared with whites; the report laid the foundations for action to eliminate these disparities through health education, promotion, and access to health care. One of the most significant outcomes of the 1985 Report of the Secretary’s Task Force on Black and Minority Health, also known as the Heckler Report, was the creation of the Office of Minority Health in 1986, with the mission “to improve the health or racial and ethnic minority populations through the development of health policies and programs that will eliminate health disparities.” The Heckler Report called health disparities among minority groups an affront both to our ideals and to the ongoing genius of American medicine [4, 5].

Thirty years after the Heckler Report was released, African Americans still endure unacceptable health disparities and lack the power over policy and actions that could make the changes to eliminate such disparities. In this paper, we review the scientific and “gray” literature on the health status of African Americans, using PubMed and government and non-government sources. Our literature search was focused on past reviews and reports and is not a comprehensive review of recent scientific research on African American health, but a review of topics that the published literature identifies as being the top priorities for improving the health status of blacks in the USA.

**Fig. 1:**
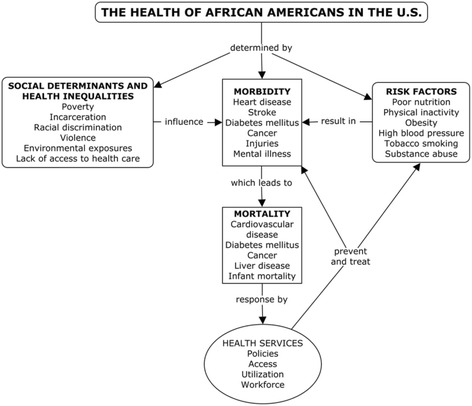
A conceptual map of the health of African Americans in the USA

This review is guided by a modified social ecological model [6, 7] that includes the social determinants of health, health disparities, main health needs, and access to health services. Recommendations are offered to help frame policies and interventions to ameliorate African American health disparities. Our conceptual model allows us to relate social (distal) determinants, with individual (proximal) determinants of health (Fig. 1). Social determinants of health include the main variables of health inequalities, namely, race, poverty, and gender. These influence health needs (morbidity, mortality, and health risks). The social response to health needs is represented by health services (policies, access, utilization, and workforce), which in turn influences health needs and risks, by hopefully resolving or improving them. Given the amplitude of our model, we delimited content to top priorities, as supported by the relevant literature. A conceptual model or framework is not intended to represent a universal truth; its purpose and usefulness is to help comprehend and transform reality. Unidirectional, static relationships depicted in a framework do not accurately reflect the historical and social world we live in—including the health of African Americans—since social determinants of health are in turn influenced by the health status of the population; also, health risks influence the social determinants of health and modulate health policies and services. Health outcomes in turn modify health risks and influence the social response by health services. The relationship between social disparities on health status of disadvantaged population has long been documented, although a direct causal pathway remains elusive [8, 9]. In this complex causal network, social determinants of health include cultural mores that influence and are influenced by the health status of populations. It should be noted that the term “health disparities” is used in this paper indistinctly from “health inequalities”; the former being any imbalances in health burdening a particular subgroup of the populace [10] and the latter understood as unjust, unnecessary, and avoidable differences affecting mostly racial/ethnic, gender, and socioeconomic vulnerable groups [11] While these terms can be semantically and conceptually distinguished, it is beyond the scope and space of this paper to do so. Also, the terms “black or African American” are used interchangeably to denote individuals that self-identify as such, as used by the US Census [12].

The following sections present the main components of African American health, as outlined in our conceptual framework: social determinants of health and health disparities, health needs (morbidity and mortality), health risks, and health services. Special emphasis is made on mental health and criminal and incarceration issues.

### Social determinants of health and health disparities

In this section, we present the main social determinants of health disparities, namely, racism, poverty, education, housing, access to healthy foods, environmental exposures, violence, and criminal justice.

In 2014, African Americans numbered approximately 42.3 million, accounting for 13 % of the US population. About 55 % of them live in the Southern states. New York State has the highest number of blacks (3.8 million), while the highest percentages are seen in the District of Columbia (50.6 %) and Mississippi (38.2 %) [13].

Racism is defined as “a belief that race is the primary determinant of human traits and capacities and that racial difference produce an inherent superiority of a particular race”^1^. It is well documented that race is a factor in health disparities that is not moderated by age, sex, and level of education [14]. Virtually, every factor considered in this document is impacted by racism. For African Americans in the USA, racism is a systemic, organized social and cultural phenomenon that, through exclusion, prejudice, and discrimination, is a cause of social and health disparities, manifested as both distal and proximal factors affecting health, for which measurements cannot always be defined [14].

Socially, racism is correlated with substandard employment, housing, education, income, and access to health services; associated risks include occupational hazards, exposures to toxic substances and allergens in the home, low-quality schooling, lack of availability of healthy foods, easy access to illicit drugs and alcohol, violent neighborhoods, and environmental exposures. Individually, racism exerts its deleterious effects through negative cognitive and emotional phenomena leading to psychopathology and morbidity, as posited by McEwen’s Allostatic Load Model [15, 16]. This model proposes that daily stressful life events diminish coping mechanisms as well as genetic makeup—through epigenetic effects—damaging immune, hormonal, physiological, and neuronal systems from cradle to grave [17, 18].

Thirty percent of African Americans believe that their health is dependent upon fate or destiny and only about 50 % feel that health is a high priority. Reception and utilization of health information are well-known major factors in disease prevention [19].

Fewer blacks graduate from high school (72.5 %) than do non-Hispanic whites (87.2 %) [20], and more whites than blacks earn a bachelor’s degree (32.5 vs. 18.6 %). As of February 2016, unemployment rates were twice as high for blacks (8.8 %) than for whites (4.3 %) [21, 22].

Poverty is a prime predictor for lacking basic human essentials including adequate clean water, nutrition, health care, education, clothing, and shelter [23]. African Americans are the poorest ethnic group in the USA. They have had the lowest median household income in the USA for the past 50 years: in 2014, measured at $35,398, compared to $53,657 for all races, and $74,297 for Asians [24]. Although African American income peaked in 2000, it has been declining ever since. Poverty is highly correlated with poor health outcomes and increased morbidity and mortality. Heart disease, diabetes, obesity, elevated blood lead levels, and low birth weight are all more prevalent among poor individuals.

Many factors in the physical environment significantly influence the health of all populations including weather, topography, air quality, and vegetation. Many other human-made influences also affect health and contribute to health disparities, but seldom receive adequate attention. The quality of housing affects health, and African Americans live in some of the country’s lowest quality housing. Asthma is related to poor housing, and African Americans are disproportionately affected from asthma. Segregated housing is correlated with a significant increase in cardiovascular disease (CVD), and African Americans live in the most segregated conditions [25]. Location is also a health determinant, and African Americans live in the poorest neighborhoods with the highest rates of homicide. Persons who live in poor neighborhoods are also much less likely to gain the benefits of exercise because of safety concerns.

Transportation is often a problem in poor communities, presenting obstacles to accessing health care services, especially preventive care, until emergencies arise.

Access to healthy foods is also a frequent problem in poor African American communities. “Food deserts” describe neighborhoods without easy access to supermarkets that sell fresh produce and other healthy foods. Black neighborhoods have significantly fewer supermarkets than white ones. Several studies also document that the food that is available in poor black neighborhoods is less fresh and of lower quality. In contrast, alcohol outlets are much more numerous in black neighborhoods. It is not surprising that rates of obesity and diabetes are highest in poor black neighborhoods [26].

Black people are significantly more likely to reside near sources of air pollution and a greater distance from air quality monitoring sites. African Americans are more likely to live in a neighborhood in close proximity to a Superfund^2^ toxic waste site. Such location has a broad negative health impact. In these neighborhoods, hospitalization for diabetes is increased; there are many adverse pregnancy outcomes—congenital heart defects, nervous system defects, low birth weight, renal dysplasia, etc. Childhood cancers are also increased in these settings. There is a 20–25 % increase in congenital anomalies among infants born in these communities [27].

Violence is also a major determinant of health disparities. It is a major cause of injury, disability, and premature death. Black male adolescents are six times more likely than whites to die of homicide, and firearms are the primary method [28]. In 2009, black males accounted for 60 % of injuries due to firearms compared to 8 % for whites [29]. There is a very significant lifelong inequity in exposure to violence for blacks vs. whites. Young black males are four times more likely to die from a gunshot than their white peers.

In 2014, African Americans accounted for 13 % of the US population but over 57 % of the victims of homicide by firearm. Firearm homicide was the leading cause of death for African American males ages 15–34, and the third leading cause of death for Latino males in the same age group (and would be second if combined with suicides in which firearms were used). Firearms were used in over 91 % of homicides of African American males ages 15–34 and in 81 % of homicides of Latino males in this age group [30]. It must also be noted that black children are twice as likely to witness domestic violence and 20 times more likely to witness a murder than white children [31]. They are also more likely to suffer maltreatment.

There are currently more than 2.3 million people housed in the nation’s 1719 state prisons, 102 federal prisons, 2259 juvenile correctional facilities, 3283 local jails, and 79 Indian Country jails as well as in military prisons, immigration detention facilities, civil commitment centers, and prisons in the US territories [32]. Approximately 50 % of all inmates in US jails and prisons are black, despite the fact that they represent less than 13 % of the population. One in six prisoners has a diagnosable mental illness. This population also suffers from infectious and chronic disease at rates that are four to ten times higher than for the total population, including a rate of HIV infection that is 13 times that of the total population [33]. Not only do prisoners come from disproportionately poor populations, but a lack of adequate healthcare has been well documented in many US prisons and jails, despite the fact that this population has a constitutional right to health services.

The number of women in prisons has been growing steadily from approximately 17,000 in 1980 (10 per 100,000 women) to 120,000 today (70 per 100,000). Black females are imprisoned at a rate nearly three times as high as white females, and seven out of ten imprisoned women have minor children.

The impact of incarceration on the family is devastating. One of every 15 black children has an incarcerated parent, compared to one of every 110 white children. Research has shown that children of incarcerated parents are six times more likely to be incarcerated themselves during their lifetimes [34]. More research must be done to improve our understanding of the long-term impact of this reality.

Because the average prison term is less than 2.5 years, about 95 % of inmates will be released and will bring their health issues back to their communities with them. Many of those released do not have health insurance, and in many states, are not eligible for Medicaid. There is a scarcity of rehabilitation programs for these individuals and inadequate attention to the resumption of basic rights such as voting. It is not surprising, therefore, that 30–50 % of former prisoners become homeless after release. Despite the fact that correctional facilities provide an opportunity to reach groups often not reached by the health and social service systems, it is, instead, a major risk factor for lifetime poor health [35].

### Major health needs of African Americans

The measures commonly used to determine the health of populations and subgroups all tell the same story. In the USA, African Americans are the least healthy ethnic group. In looking back at the progress made toward eliminating health disparities in Healthy People 2010, disparities between the non-Hispanic black population and the population with the best rates increased for 34 objectives (13 %). Ten of those objectives were for death rates: neonatal and postneonatal deaths, adolescent deaths, firearm-related deaths and homicides, diabetes-related deaths, and deaths due to HIV infection, coronary heart disease, stroke, and cardiovascular disease among persons with chronic kidney disease.

A decrease in disparity was observed for 29 of the objectives (11 %); most of the decreases were found in objectives addressing sexually transmitted infections and HIV. Below, we present some of the main health indicators of health needs for African Americans [36].

### Age-adjusted death rates

Blacks had the highest age-adjusted death rate of any ethnic group in 2013 (1083.3 per 100,000 standard population for black males vs. 876.8 100,000 for white males, the second highest). The rate for the total population was 731.9 per 100,000 population making the black male rate 48 % higher than the total.

### Life expectancy and mortality

In the USA, from 1980 to 2014, life expectancy at birth increased from 70.0 to 78.8; 76.4 years for males and 81.2 years for females. Life expectancy at birth for blacks is 75.2 years; 72 years for males and 78.1 years for females. The gap in life expectancy at birth between blacks and whites decreased from 5.2 years in 2004 to 3.4 years in 2014. Between 2004 and 2014, the mortality rate among 45–54-year-old black males decreased 28 % from 933.3 to 671.8 deaths per 100,000 population, while the rate for white men did not change (511.2 deaths per 100,000 population). Among 45–54-year-old black women, the mortality rate decreased by 18 % while it increased 11 % for white women [37, 38].

### Years of Potential Life Lost (YPPL)

Overall, African Americans remain the least healthy ethnic population. There seems to have been marked improvement in this picture by 2010. African Americans ranked first in only four of the top 10 causes, but the listed causes had changed. Poisoning was added as a new cause and cirrhosis of the liver edged out HIV and diabetes (for both of which African Americans were number one) for the tenth spot.

### Infant mortality rates

Perhaps black infant mortality provides the most transparent view of black health. It has always been at least 2.5 times greater than the white rate since data have been recorded. The total rate for all ethnic groups has declined steadily since reporting was initiated, but the disparity between black and white infant mortality rates persists. Interestingly, there was a pause in the decline for all ethnic groups from 2000 to 2005. This pause was due mostly to the increase in “preterm” and “very preterm” births by minority mothers. In 2005, the infant mortality rates were 6.86/100,000 for all births, 5.76/100,000 for whites, and 13.6/100,000 for blacks.

The decline recommenced for the period 2005 to 2010. While the decline for the total population was 12 % during this period, the black infant mortality declined 16 %—the greatest decline for any ethnicity [39]. However, the total US infant mortality rate was 5.96 infant deaths per 1000 live births in 2013, and that for African American infants was 11.1 per 100,000 live births despite the recent progress [40]. The low birth weight (LBW) level was 6.98 % for non-Hispanic white women and 13.08 % for non-Hispanic black infants in 2013. And, in 2013, the rate of preterm deliveries was 1.6 times higher for African American women. In 2014, low birth weight and preterm births before 37 weeks gestation were the highest among black women, 13.17 and 11.1 %, respectively [37, 41].

In 2002, blacks trailed whites in women receiving prenatal care in the first trimester (75 vs. 89 %) [42]. In 2008, only 59.1 % of African American women giving birth to live babies had any prenatal care vs. 72.2 % of white women. 11.5 % of African Americans who received any prenatal care commenced in the third trimester [43].

Black women were also more likely to report not receiving advice from their prenatal care providers about smoking cessation and alcohol use. There was also less counseling regarding breast-feeding although the difference was not significant in this study [44]. As noted above, the gap between infant mortalities declined slightly between 2007 and 2010, but black infant mortality remains more than 200 % that of whites. Looking at the youngest mortalities, black infants have a significantly higher neonatal and post-neonatal mortality than any other ethnic group [45].

Most sources indicate that more than 75 % of all infants receive well baby care. Therefore, the differences in mortality are due to factors which have already made their impact at the time of birth, such as the health status of the parents at conception, genetics, and environment [46].

### Chronic diseases and homicide

Over recent decades, four main causes of morbidity stand out: heart disease, diabetes, cancer, and homicide. The black population displayed a larger decrease in death rates for heart disease, cancer, and HIV disease accounting for the narrowing gaps. Additionally, there was a larger decrease in unintentional injuries in black males.

Heart disease is the leading cause of death for most Americans; 46 % of African Americans older than 19 years of age suffer from cardiovascular disease. According to the CDC, leading risk factors for heart disease and stroke currently are high blood pressure, high cholesterol, diabetes, current smoking, physical inactivity, and obesity. Individuals with two or more of these factors are at a higher risk for stroke and heart disease. In 2003, the prevalence of two or more of these factors was highest in African Americans, and heart disease among African Americans has been the first or second cause of Years of Potential Life Lost in the USA ever since these data have been kept. This disparity is not surprising given that African Americans had the highest prevalence of hypertension from 2007–2010; in 2011–2014, black men and women aged 20 years and older continued having the highest prevalence of hypertension (42.4 and 44 %, respectively) [37]. African American women had the highest prevalence of obesity during this period, and African Americans have had the highest rates of diabetes since the data have been collected. According to a 2014 report, blacks have a “1.3-times greater rate of non-fatal stroke, a 1.8-times greater rate of fatal stroke, a 1.5-times greater rate of death attributable to heart disease, and a 4.2-times greater rate of end-stage kidney disease” [47].

### Cancer

When looking at all cancers, African Americans were the group most heavily impacted in 2012. Incidence rates were highest in the black population (554.5/100,000) and so was the death rate (253.9/100,000). Among women, however, the overall incidence rate for cancers is not the highest among ethnic groups, but the death rate is.

**Table 1:** Prostate cancer incidence and death rates

Racial/ethnic group	Incidence	Death
All	168.0	27.9
African American/Black	255.5	62.3
Asian/Pacific Islander	96.5	11.3
Hispanic/Latino	140.8	21.2
American Indian/Alaska Native	68.2	21.5
White	161.4	25.6

Statistics are for 2000–2004, age-adjusted to the 2000 US standard million population, and represent the number of new cases of invasive cancer and deaths per year per 100,000 men [110]

A health disparity among women is best illustrated by breast cancer. While the incidence rate per 100,000 was lower for African American women (118) than for white women (133) and the total population (127), the death rate was highest among African American women at 33.8 per 100,000 vs. 25.5 for all women.

For African American males, the greatest cancer disparity is prostate cancer. For the period 2000–2004, the incidence was 255.5 per 100, 000 vs. 161.4/100,000 for white males and 168/100,000 for the total population (Table 1) [48].

### Homicides/interpersonal violence

When reviewing health disparities, homicides are always the cause of death with the largest ethnic disparity. It alternates with CVD as the greatest cause of YPLL for African Americans. From the period 1999–2002, black males died from homicide at ten times the rate of whites. In 2013, the age-adjusted homicide rate for blacks was 18.7 deaths per 100,000 population. This figure was three times greater than the rate for any other ethnic group. While black males had the highest rate, black females had a higher rate of homicide deaths than any other females, and intimate partner homicide was a major factor [49].

Homicide is the absolute measurement of violence, revealing the unquestionable ethnic disparity. However, violence affects African Americans in many other ways. In 2013, higher rates of aggravated assault, child maltreatment, and fights among high school students were reported. In 2011, African American women reported higher rates of experiencing rape and physical violence by an intimate partner [49].

### Mental and behavioral health

National surveys show lower rates of mental disorders for blacks and Hispanics (16.9 %), as compared to whites (19.3 %) [50]. Yet, more blacks suffered serious psychological distress than whites in the previous year (6.9 vs. 4.4 %, respectively). That is to say, black people endure more intense and frequent mental and behavioral health issues than their counterparts, at least in part related to poverty and exposure to racism and discrimination, both of which disproportionally affect minorities [51–53].

Yet, the proportion of youth who had serious thoughts of suicide was 40 % lower among black youth 18–25 years, compared to their white counterparts (5.0 vs. 8.2 %), and this highlights the resiliency needed for blacks for everyday survival.

It has been shown that blacks receive fewer medication due to racial bias, low income, and insurance status. A study showed that blacks with depression and without insurance receive fewer antidepressants than those insured; even among the insured, they received fewer medication than whites [54]. Another study found that fewer blacks receive opioids at discharge from emergency department visits due to back and abdominal pain, compared to whites [55]; also, it has been reported that substance abuse treatment centers serving higher percentages of minority populations prescribe fewer selective serotonin reuptake inhibitors (SSRIs) than centers serving fewer minority clients [56].

With respect to tobacco, African Americans have lower rates of use than whites. For example, the 2013 NSDUH estimated that 27.6 % whites and 26.6 % blacks used tobacco products in the past month [57]. However, analyses have identified a crossover at age 29, whereby blacks end up with higher rates of tobacco use than whites, largely which were traced back to differences in educational attainment and marital status [58]. Almost 30 years ago, the CDC concluded a report on racial disparities that reducing tobacco could lead to reducing wide health disparities in the following terms.

The reduction of cigarette smoking in the black population is one of the most important, immediately available options for reducing the wide disparities between the health status of minorities and that of whites [59].

Yet, the research discussed by the Surgeon General on the 50th Anniversary of the first Surgeon General Report on Tobacco showed that blacks continue to suffer a disproportionate burden of tobacco-related mortality and morbidity [60].

Obviously, much remains to be achieved when it comes to addressing the needs of the poorest communities in America, which are also those who suffer the highest rates of tobacco use and tobacco-related consequences. Community-Based Participatory Research (CBPR) is a promising approach to help overcome the lack of adequate smoking cessation programs for minority and underserved populations, such as the implementation of community-based smoking cessation interventions that are peer-based and place emphasis on behavioral change training and social support, along with the use of nicotine replacement therapies and strategies toward stress management [61]. These and other efforts take advantage of federal and state-funded quit-lines that offer free counseling and nicotine replacement therapy to those interested in quitting tobacco. However, it is also necessary to acknowledge that the high cost of effective medications has been an important barrier to quitting tobacco among blacks and other minority groups.

The situation regarding drug use such as marijuana and cocaine is no different. Blacks have a slightly higher rate of past-month use of marijuana as compared to whites (10.3 vs. 8.7 %, respectively). Whites and blacks have similar rates of past-month use of cocaine (0.6 %). However, the consequences of drug use disproportionally affect blacks. For example, blacks have higher rates of DSM-IV substance abuse/dependence to illicit drugs than whites (4.1 vs. 2.4 %, respectively, in the past year), but not of alcohol abuse/dependence (6.0 vs. 6.5 %, respectively). Only one third to one fourth of people in need of addictions treatment got it, at least as reflected in the latest national survey of drug use. The impact of substance use and mental health problems is evident in other social domains. For example, in 2014, there were 619,809 arrests due to marijuana possession (39.7 % of all drug arrests) (http://www.drugwarfacts.org/cms/Marijuana#Total). Blacks were almost four times more likely than whites to be arrested for marijuana, in spite of the fact that both groups have relatively similar use rates as shown earlier [62].

About two out of three people with any mental illness also had alcohol or drug dependence (66.8 %). Yet, among blacks, only two out of three people with Major Depressive Episode (MDE) received any form of treatment (64.6 %), and one in four people with severe MDE and impairment did not receive treatment (26.3 %). Overall, 55.3 % of Americans with an unmet mental health need did not receive treatment or counseling in the past year, reporting that they “could not afford the cost,” 25.5 % “did not know where to go,” and 24.6 % thought they “could handle the problem without treatment” [50]. While we cannot break down these data to analyze possible racial and ethnic differences, it is important to also consider that stigma has been major factor in blacks not receiving mental health care and may further complicate the effect of other healthcare barriers [63–66].

In sum, the data depict a rather complex picture in which blacks generally have similar if not lower incidence rates of mental disorders and substance involvement than whites, but at the same time suffer higher prevalence of serious mental health and legal problems, with devastating effects. The difference between lower incidence and higher prevalence derives from longer duration, given lower access and utilization of healthcare services, lower quality of healthcare services, and worse complications of comorbidities for minority and underserved populations, among others [56, 67–72]. In convergence, the greater impact of mental health problems for blacks stems from structural factors that include poverty, racism and discrimination, and culture [73–75], such that the stress caused by the interaction of poverty, inequality, and discrimination affects blacks above and beyond of the effect on other non-minority populations. Clearly, several barriers need to be lifted if we are to make progress in this area, beginning by providing expanded true access to healthcare services including transportation, availability of culturally friendly services, and the establishment of mechanisms that will avoid stigmatizing clients and ensure confidentiality of the data [51].

Unfortunately, a growing body of research documents that physician bias leads to a lower quality of care for CHD based on patient’s race/ethnicity, sometimes patient gender, and his/her socioeconomic status [76].

### Health care services: policies, access, and utilization

In this section, we review the social response to health needs of blacks, as implemented through policies and programs, as well as issues of access and utilization. The role of an educated and culturally sensitive workforce is highlighted.

In 2003, the Institute of Medicine released *Unequal Treatment: Confronting Racial and Ethnic Disparities in Health Care*, a comprehensive review of disparities in healthcare treatment. The attitude expectations and behaviors of providers and patients were examined. Conscious and unconscious differences in treatment based on ethnicity, socioeconomic status, and gender were reviewed. This document reviewed the biases, stereotypes, and communication obstacles impacting the interaction of providers and patients and their utilization of the healthcare system. Findings in the study concluded that:

“Bias, stereotyping, prejudice, and clinical uncertainty on the part of healthcare providers may contribute to racial and ethnic disparities in healthcare”; and that, “a small number of studies suggest that racial and ethnic minority patients are more likely than white patients to refuse treatment” [77].

In recent years, blacks have had worse access to care than whites for about half of access measures used. During the first half of 2014, the percentage of adults ages 18–64 without health insurance decreased more quickly among blacks and Hispanics than whites, but differences in insurance rates between groups remained.

In March of 2010, President Obama signed in to law the *Patient Protection and Affordable Care Act* (ACA) to increase the quality and affordability of health care by increasing access to insurance. This plan aims to increase healthcare protection by expanding coverage, holding insurance companies accountable, decreasing healthcare costs, allowing provider choice, and enhancing the quality of care [78].

About eight million African Americans obtained access to expanded preventive services, and nearly eight million African Americans with a preexisting health condition became able to obtain coverage. Since the first quarter of 2015, the uninsured rate dropped by 9.2 percentage points among African Americans in the 18–64-year-old age group. Approximately 5.7 million young adults gained coverage from 2010 through March 4, 2015, dropping the young adult uninsured rate by 7.4 % [79, 80].

The ACA is changing the funding of hospitals from a system based on quantity of patients and procedures to one focused on quality of care—value instead of volume. Now, many of the responsibilities that have traditionally been those of public health are incorporated into the “Community Health Plan” of the hospital and expansion of Medicaid. This change in the basic protocol of healthcare delivery in the USA offers a significant opportunity for African Americans and other under-represented and minority groups to insert themselves into the health care infrastructure [81].

However, not all states have undergone Medicaid expansion under the ACA, with negative consequences for access and the health status of minorities and the poor. Texas and Mississippi—states with higher percentages of black populations—are among the 17 that have rejected Medicaid expansion [82]. According to the Kaiser Foundation, 40 % of eligible black adults live in states rejecting Medicaid expansion and are two-fold more likely than whites and Hispanics to remain uninsured [83].

Also, a study showed that, in states not expanding Medicare, low income adults aged 18–64 were more likely to be black and reside in rural areas than in states expanding Medicare; also, they were less likely to have a usual source of care and use preventive services (dental checkups, routing checks, flu vaccinations, and blood pressure checks) [84].

### Health workforce

An educated and informed black population will use health care services more effectively. Forty percent of African Americans have limited reading skills [85]. Health literacy is one’s ability to obtain, process, and understand basic health information and services. This skill is necessary to make appropriate health decisions. Good health literacy requires the reading, analysis, and decision-making skills to make appropriate health decisions. Lack of health literacy skills is considered a cause of health disparities, and disparities by both race and educational status when health literacy are taken into account [86]. People with poor health literacy have problems communicating with their health providers, reading instructions on medicines, and completing medical and insurance forms.

In 2012, blacks were 13.6 % of the “working age population” but were not 13 % of any of the major health professions. In the present, only 5.3 % of active physicians are black, and that is true for 10 % of nurses. Oral health remains a major issue for African Americans, but only 3 % of dentists are black.

As we look more broadly at clinical providers, we see that only 5.2 % of advanced practice registered nurse (APRN), 8 % of physician assistants (PA), and ~5 % of pharmacists are black. Expanding the view even further, only 4 % of occupational therapists and speech therapists are African American.

Historically Black Colleges and Universities (HBCUs) have been a major educational resource for African Americans since the end of slavery almost 150 years ago. The primary mission of HBCUs is to educate black Americans. There are currently 100 HBCUs, and about 30 % of the BA degrees awarded to African Americans annually are produced by the 89 4-year HBCUs.

In 2010–2011, blacks earned 85 % of the 33,000 bachelor’s degrees conferred by HBCUs and 73% of the master’s degrees. From another perspective, HBCUs awarded 35 % of the bachelor’s degrees to blacks in the USA in 1976–1977. This figure dropped to 16 % in 2010–2011. Facing the increasing need for health professionals, there has been a significant expansion of health training programs across the USA. It is stunning to see that health programs in HBCUs have not shared in this growth. In 2007, only 5 % of full-time faculty members at higher education institutions were black [87]. In 2011, non-black students made up 19 % of enrollment at HBCUs. In 2013, only 60 % of nurses trained in HBCUs were African American [88].

### The future

This section presents the key messages the authors would like to convey regarding social determinants of health and health disparities, health needs, and healthcare policy and services, to improve the health of African Americans in the USA

Given all that has been detailed, it is obvious that there is much to be done if we are ever to achieve health equity or eliminate health disparities in the USA and assure good health to the African American population. A goal of Healthy People 2010 was the “elimination” of health disparities. It was not achieved for African American people [89]. The current picture is clear; the greatest health disparity between the total US population and any ethnic group is found in African Americans.

As stated in the introduction, racism may be the most important phenomenon underlying black health disparities, exerting its ominous effects through institutionalized, systematic stigma and exclusion. As we have shown, health disparities for blacks are racial disparities; social and gender disparities are interwoven and magnified to render blacks the least healthy of all groups. Historically structured racist practices and institutions are further reproduced by white-majority policymakers, decision makers, administrators, educators, and healthcare providers. Addressing “health disparities,” “cultural competence,” and “racial bias” at the individual level through healthcare services misses the social, institutional, and organizational levels underlying health disparities among blacks. At the individual level, this focus is translated into insufficient allocation of resources to black communities and populations [90].

Poverty, low education, unemployment, violence, insecurity, and environmental exposures contribute to poor reproductive health and birth outcomes among black women [90]. These factors affect the woman and her family at multiple levels: low access to healthy foods, inadequate access to preventive and antenatal healthcare, intimate partner violence [91], distrust of the justice and police system [92], unhealthy behaviors, substance abuse [93], and stress [94].

A greater proportion of black children are born and live in this social, environmental, and culturally deprived environment; thus, they grow and develop unequally—socially, psychologically, and healthwise, throughout the lifespan [95].

Research into minority and black health issues has been found to be both insufficient and biased [96].

The systemic nature of racism as a cause of health disparities must be counteracted by equally systemic measures, through social programs, economic investment, criminal system reform, decreased segregation in positions of institutional power, more inclusive research and appropriate funding of public agencies, healthcare institutions, and HBCUs [90, 97]. Further implementation and expansion of the Affordable Care Act should result in improved health outcomes for black populations [98].

Of course, addressing the wide ranging consequences of poverty is a social problem that all those working for health equity must attempt to redress. Although there has been significant progress in assuring healthcare for the poor with the ACA and other programs, health institutions must not pretend that adequate healthcare is available to all. The care that is provided to all must be of the highest quality, not only technically but ethically. Physicians and public health professionals, black and otherwise, must stand for racial and social justice [99]. Proactive efforts must be taken throughout health systems to eliminate the conscious and unconscious differences in the quality of care currently provided in all aspects of medical practice. These efforts must be directed at the practice of all health providers and the functioning of all systems [100].

Public health should take the lead in advocating for and providing the expertise to assure that inadequacies in physical and social environments do not harm African American populations. In the physical environment, priorities include informing at-risk populations of the impacts of their unhealthy environments, assurance of good housing and transportation, and documenting the location and impact of toxic waste; these interventions should be approached through cross-sector collaborations [101].

The health of persons under the control of judicial and incarceration systems may be one of the highest priorities in the social arena. A concentrated effort to educate and train justice system administrators and staff in the basic principles of health care is necessary, and the provision of health services should be overseen by an unbiased body which is independent of the justice system. It has been demonstrated that healthcare systems based outside prison walls can provide excellent healthcare to inmates and eliminate the barriers which prevent returning convicts from receiving appropriate care upon release [102].

Many strategies such as advocating for the appropriate location of supermarkets and farmers’ markets and promoting of inner city community gardens can have a significantly positive impact on the health of African Americans [103].

Addressing the problems of nutrition and food deserts should be high priorities. Diabetes, CVD, and obesity will be directly affected, while many other major health problems in the African American community will be impacted [104, 105].

Many other disparities contribute to the poor health status of African Americans. Depending on how “cause of death” is determined and how it is calculated, diabetes is often in the top 10 causes of morbidity and mortality for African Americans. The same can be said for substance abuse, lung cancer, and stroke. African Americans are over-represented when the top 10 causes of Years of Potential Life Lost are documented. Mental illness is a major problem, but much work needs to be done to develop an accurate and useful picture of the overall disparity [106].

Access to preventive, curative, and rehabilitative care must be assured to all persons including African Americans. Access is a lifelong need. Care for the potentially pregnant women is crucial and may have long-term consequences for her and her offspring. Comprehensive care for the infant, child, and adolescent is the key to their lifelong health and also their ability to function as productive and creative people. Adults often must be reminded that there are standards for healthcare from which they will benefit, and, as the population ages, access to appropriate and comprehensive care must be assured for elderly African Americans.

In order to assure care of the highest quality, proactive efforts must be taken throughout health systems to eliminate the conscious and unconscious differences in quality of care provided. These efforts must be directed at the practice of all health providers and all systems. Today, the differences are integral to virtually all health practice [107].

Education at all levels may be the most important role of health professionals. It is our responsibility to translate our knowledge of health into the language and culture of the client we are serving. Minorities are more likely to seek care from healthcare professionals of their own ethnicity. Communities are more than willing to collaborate with providers in taking on this task [108].

The development of health policy is most often the responsibility of those with no health expertise, with little representation of the black population. Without the education of health professionals who are knowledgeable of the culture of African American communities and committed to their well-being, the future of policy development is bleak [109]. HBCUs have played a major role in a variety of fields in the 150 years of their existence and are not being appropriately utilized in the training of black health professionals.

Also, the policies of health practice and health institutions that serve African Americans are most often determined by public and private sector leaders who have no health training. It is the responsibility of trained health professionals to provide the information needed to make appropriate health policy decisions and to evaluate their implementation. In addition to these factors, communities, providers, and individuals must all understand that politics is a key factor in the ongoing battle to eliminate the disparities in health outcomes in the USA that are based on racial differences.

## Conclusions

After 250 years of social segregation and discrimination, current health data confirm that African Americans are the least healthy ethnic group in the USA. Although the resources and policies to eliminate disparities exist in the USA, there has been inadequate long-term commitment to successful strategies and to the funding necessary to achieve health equity. African Americans have not been in the fiscal nor political positions to assure the successful implementation of long-term efforts; the health of African Americans has not been a priority for decision makers.

Usually, the black community is not present when strategies and programs addressing their poor health status are designed and prioritized, and planners have limited understanding of the social mores and history of the African American community. The administration of health and social organizations serving black communities is rarely in the hands of those with this knowledge and commitment.

Current mortality disparities are evident in cardiovascular disease, cancer, diabetes, and infant mortality. These causes of death may be the most visible health problems for African Americans, but they do not tell the whole story. Mental illness is the second largest cause of morbidity in African Americans, and violence in the form of homicide is the greatest cause of preventable death. High levels of poverty, lack of education, and excess incarceration further compound the poor health status of African Americans.

The USA is in the midst of a surge in training health professionals, but, for many reasons, the institutions (HBCUs) created to educate African Americans have not made much impact on advancing the health of African Americans. African Americans are under-represented in all of the professions responsible for the provision of intimate physical, mental, and social care.

All health providers should be required to obtain regular training and refreshing in the provision of equitable care; this includes providers of color. Training of young people of color in the health professions should be viewed as an urgent national objective requiring the rebuilding of many of social development and community health programs of the past which have been virtually extinguished by lack of funds. Outreach to young people of color encouraging them to pursue health careers should be given a much higher priority. The role of HBCUs in the preparation of young populations for health careers must be strengthened.

It is evident that focusing on health risks alone is not conducive to redressing health disparities among African Americans, given that structural factors primarily underlie their poorer health outcomes and shorter lifespans. Tackling the social determinants of health, from poverty to the built environment, racial discrimination, violence, and incarceration, is likely to elicit greater effects on black health than risk reduction programs. Even though the ACA has expanded access to African Americans, medical care for people with unhealthy lifestyles and social and cultural barriers to access will have limited effects on reducing health disparities of African Americans in the USA.

## Footnotes

1 Merriam-Webster’s Collegiate Dictionary, Tenth Edition, 1998

2 Superfund sites are hazardous waste cleanup sites managed by the US Environmental Protection Agency since the early 1980s. They pose potential threats to human health and the environment. https://www.epa.gov/superfund
